# Imaging Conditioned Fear Circuitry Using Awake Rodent fMRI

**DOI:** 10.1371/journal.pone.0054197

**Published:** 2013-01-22

**Authors:** Nichola M. Brydges, Heather C. Whalley, Maurits A. Jansen, Gavin D. Merrifield, Emma R. Wood, Stephen M. Lawrie, Sara-Madge Wynne, Mark Day, Sue Fleetwood-Walker, Douglas Steele, Ian Marshall, Jeremy Hall, Megan C. Holmes

**Affiliations:** 1 Centre for Cardiovascular Science, The Queen's Medical Research Institute, University of Edinburgh, Edinburgh, United Kingdom; 2 Edinburgh Neuroscience, University of Edinburgh, Edinburgh, United Kingdom; 3 Edinburgh Preclinical Imaging, University of Edinburgh, Edinburgh, United Kingdom; 4 Centre for Cognitive and Neural Systems, University of Edinburgh, Edinburgh, United Kingdom; 5 Strategic Transactions Group, Bristol-Myers Squibb Company, Wallingford, Connecticut, United States of America; 6 Medical Research Institute, University of Dundee, Dundee, United Kingdom; IGBMC/ICS, France

## Abstract

Functional magnetic resonance imaging (fMRI) is a powerful method for exploring emotional and cognitive brain responses in humans. However rodent fMRI has not previously been applied to the analysis of learned behaviour in awake animals, limiting its use as a translational tool. Here we have developed a novel paradigm for studying brain activation in awake rats responding to conditioned stimuli using fMRI. Using this method we show activation of the amygdala and related fear circuitry in response to a fear-conditioned stimulus and demonstrate that the magnitude of fear circuitry activation is increased following early life stress, a rodent model of affective disorders. This technique provides a new translatable method for testing environmental, genetic and pharmacological manipulations on emotional and cognitive processes in awake rodent models.

## Introduction

There is a clear requirement for improved translation of preclinical neuroscience research into clinical impact. One particular area of need is the development of measures of neural activation that can be translated directly from animals to humans. Functional Magnetic Resonance Imaging (fMRI) provides a system-wide measure of neuronal activation which is non-invasive, repeatable and lends itself to paradigms translatable across species. Until now however, rodent fMRI has been largely limited to anaesthetized animals [1], [2] or animals at rest or responding to an innate stimulus [3]–[6], and has not been applied to the study of learned behaviours in awake animals, limiting its use as a translational tool. Here we have used fMRI to assess emotional function in awake rodents with the aim of generating translatable methods for investigating the aetiology and treatment of neuropsychiatric disorders.

In order to develop awake rodent fMRI of a learned task, we focused on Pavlovian fear conditioning. Pavlovian fear conditioning produces robust behavioural and physiological responses and has been shown to elicit amygdala activation in human imaging studies [7]–[9]. Furthermore abnormal fear conditioning and altered amygdala activation have been implicated in a number of human neuropsychiatric conditions including affective disorders, post-traumatic stress disorder (PTSD) and schizophrenia [7], [10]. The development of rodent imaging models of fear conditioning is therefore of utility for studying translational models of these conditions. Early life stress (ELS), known to increase fear and anxiety responses in adulthood, has been extensively used as an animal model of human affective disorders [11]–[13]. Furthermore ELS in humans is associated with later affective disorders and increased amygdala activation in adulthood [14]–[17]. Here we therefore report both the development of awake rodent fMRI imaging and its modulation by ELS.

## Materials and Methods

### Animals

32 male Lister Hooded rats (Harlan, UK) weighing 260 g ±20 g were housed in small groups (4–5 per cage) with water and chow available *ad libitum* in a humidity (45–60%), temperature (21°C) and light (on 07.00–19.00 h) controlled environment. Prior to experimentation, all animals were handled daily for 14–21 days. All studies were carried out in strict accordance with local ethics guidelines (approved by The University of Edinburgh Ethical Review Committee), the UK Home Office Animals (Scientific Procedures) Act 1986 and under a personal Home Office license.

### Acclimitisation of Animals to the MRI Scanning environment

Prior to scanning, animals were acclimatised to the restraint apparatus and scanning environment on days 1 and 3 using a mock MRI scanner [18]. This procedure produced stable physiological responses within the normal range for adult rats (Table 1). Acclimatisation was carried out in a purpose built mock scanner, with recordings of scanner noise played through speakers in the apparatus at a level equivalent to that measured in the scanner itself. All animals were placed in the mock scanner within a rodent restraint apparatus in order to minimize motion artefacts during subsequent MRI scanning (InsightMRI, Worcester, MA). The restraint procedure is described in King et al. [18]. Briefly, a plastic semicircular headpiece with blunted ear supports that fit into the ear canals was positioned over the ears. The head was placed into a cylindrical head holder with the animal's canines secured over a bite bar and ears positioned inside the head holder with adjustable screws fitted into lateral sleeves. An adjustable surface coil built into the head holder was pressed firmly on the head and locked into place. The body of the animal was placed into a body restrainer. The head-piece locks into a mounting post on the front of the chassis. This design isolates all of the body movements from the head restrainer and minimizes motion artifact. Rats were lightly anaesthetized using 1–2% isoflurane in air while being placed into and removed from the restraint apparatus and then allowed to recover consciousness thereafter.

**Table 1:** Heart rate levels obtained during preliminary testing using a mock scanner.

Day	Heart rate (beats per min)
1	300+/−27 S.E.
3	284+/−8.7 S.E.
5	311+/−9.8 S.E.

Data Mean ±SEM, n = 6 rats. Previous telemetry evidence suggests a resting heart rate of around 300–380 bpm in adult rats.

### Animal preparation and conditioning protocol

**Figure 1:**
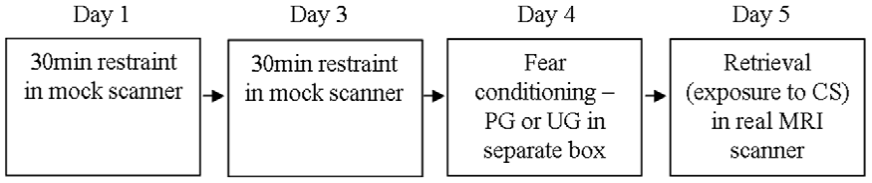
Training and scanning procedure. Animals were acclimatised to the scanning procedure using a mock scanner, conditioned and then tested during retrieval in the scanner 24 h later.

Animals were subsequently conditioned to a fear-associated cue on day 4 in a separate and distinct context, and were scanned in the real MRI scanner on day 5 (Figure 1). The conditioning apparatus comprised a rat shock chamber (30 cm ×25 cm ×32 cm), and the conditioned stimulus (CS) was a 10 s flashing light (10s of 5 Hz maximum intensity flashes, 50/50 duty cycle). The unconditioned stimulus (US) was a 0.5 s, 0.5 mA foot shock delivered through the bars of the floor. Fear conditioning was conducted on experimental day 4, allowing retrieval of the conditioned response in the scanner 24 h later. Animals were trained in paired and unpaired groups (PG (n = 14) and UG (n = 10) respectively). The PG received 5 presentations of the CS, each co-terminating with the US. These pairs were presented at intervals of 5±1min for a total of 25 min. The UG received 5 presentations of the CS (intervals 5±1 min) with 5 presentations of the US in a pseudorandom order (explicitly avoiding any pairing) over 25 min.

**Figure 2:**
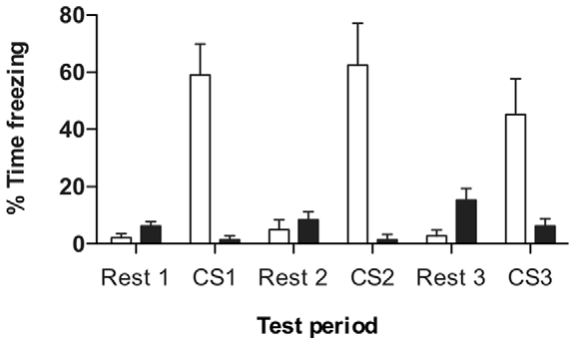
Behavioural response to the fear conditioning paradigm. Animals were trained with paired CS-US presentations (paired group (PG), open bars; n = 6) and unpaired CS and US presentations (unpaired group (UG), black bars; n = 6) and freezing behaviour was assessed 24 h later in a separate context. A two-way ANOVA revealed that PG spent a greater proportion of time freezing than UG animals during all three stimulus presentations (*P*<0.01). Data is represented as mean ± SEM.

We confirmed that conditioned fear responses are produced to subsequent presentations of the CS in PG animals but not in UG animals in a separate behavioural experiment using conditioned freezing as a dependent variable. 12 rats were conditioned using the above fear conditioning protocol. 6 experienced the PG procedure, 6 the UG procedure. Rats were conditioned on day 1, and placed into a novel context (to reduce contextual freezing) on the following day for retrieval. The novel context was a chamber (30 cm ×25 cm ×32 cm) with the walls and floor decorated with coloured plastic shapes, to distinguish it contextually from the training chamber. During retrieval, 12 blue LED lights, mounted in the ceiling, were flashed (5 Hz maximum intensity flashes, 50/50 duty cycle – identical to the training lights) for a duration of 2 min, followed by a rest of 2 min, and this was repeated twice more. The percentage of time the animals spent freezing during all periods was calculated. Only rats that had been conditioned to associate the conditioned stimulus (CS) with the aversive shock (US) exhibited freezing behaviour upon each presentation of the CS 24 h later (p<0.01). Animals from the unpaired group did not show a significant freezing response to the presentation of CS (Figure 2).

### Image acquisition and analysis

The day after fear conditioning (day 5), rats were lightly anaesthetized, put into the restraint apparatus and placed into a 7T MRI scanner (Agilent Technologies; formerly Varian, Oxford, UK) equipped with a 400 mT/m gradient set, a 72 mm volume RF coil for transmission and a surface coil for signal reception (InsightMRI, Worcester, MA). Anaesthesia was maintained until the animal was positioned in the coil assembly in the scanner and whole-brain structural images were acquired using a Fast Spin Echo (FSE) sequence with a matrix of 256×256, FOV 30 mm (in-plane resolution 117 μm), TR = 3000 ms, Echo Train Length (ETL) 8, effective TE = 48 ms, 4 signal averages, and 22×1 mm coronal slices. Animals were then allowed to regain consciousness (respiration level >85 breaths/min) before the test paradigm and functional scanning commenced.

After a 5 min acclimatisation period, bilateral visual CS stimuli were presented in the scanner using a custom-built array of high-intensity blue LEDs fitted with a diffuser. The activation protocol consisted of 2 min periods with the LEDs flashing at 5 Hz with a 50/50 duty cycle (“on”) alternating with 2 min periods during which the LEDs were not illuminated (“off”) with a randomized starting order. A total of three CS presentations were delivered in each scanning session. Functional image acquisition used a FSE sequence with a matrix of 64×64 (in-plane resolution 469 μm), TR = 2500ms, ETL 16, and effective TE = 36ms. Each functional volume consisted of 16×1 mm slices covering the brain excluding cerebellum and took 10s to acquire. Functional scanning was run for a total of 103 volumes (17 min 10 s).

### Data analysis

SPM5 (Wellcome Trust Centre for Neuroimaging, University College London, UK) was used for image analysis. Structural and functional data for each individual animal was manually checked for artifacts. To facilitate SPM processing, images were scaled by a factor of 10 in the x, y and z dimensions to allow for differences between rat and human brain sizes. A structural study-specific rat brain template was created using 18 Lister Hooded rats based on the Karolinska template (consistent with the Paxinos Atlas space [19]). A priori tissue segments were generated for SPM5 preprocessing procedures. Functional data for each animal was realigned to the mean volume in the series, and then spatially normalized according to SPM5 co-registration procedures using each individual animal's structural scan. Finally, all realigned and normalized images were smoothed with an 8×8×8 mm full width half maximum (FWHM) Gaussian filter. Global effects were removed using a voxel level linear model of the global signal [20].

First level analysis was performed using a general linear model implemented in SPM5. At an individual subject level, the 3 on/off periods were modelled separately along with the initial acclimatisation condition. This permitted analysis of individual blocks and therefore habituation effects. Each condition was modelled by a boxcar convolved with the SPM5 canonical hemodynamic response function. Contrasts were constructed to determine (i) the first CS block responses > all ‘off’ blocks (baseline), (ii) the first CS block responses > the last CS block, and (iii) the last CS block responses > the first CS block. For second level analysis one contrast image per subject for each contrast of interest (i–iii) was entered into a second level random effects model. Within group effects were modelled using a one sample t-test. Between-group differences were investigated using a two-sample t-test. Second level images were thresholded at a whole brain cluster level of P_corrected_<0.05. Monte Carlo simulations [21] showed that this was achieved by a simultaneous requirement for a voxel level significance of P<0.05 and activation clusters exceeding 387 contiguous voxels. A region of interest (ROI) analysis was applied for the lateral amygdalae using an anatomical rat bilateral lateral amygdala mask generated using MRIcro (http://www.cabiatl.com/mricro/mricro). Further Monte Carlo simulations indicated that when only the amygdala mask was of a priori interest, cluster level P_corrected_<0.05 was satisfied by a simultaneous requirement for a voxel level significance of p<0.05 and activation clusters exceeding 56 contiguous voxels.

### Early Life Stress protocol

In order to examine the effects of early life stress on fear circuitry activation a further group of animals was subjected to a repeated variable stress paradigm between postnatal day (PND) 25–27 using previously established protocols [11]–[13]. This consisted of a 10min swim in a bucket of water on PND 25 (25 cm height, 34 cm diameter, temperature 25+/−1°C), restraint in a Perspex rat restraint tube on PND 26 (15 cm length, 5 cm diameter) for 3×30 mins with a 30 min break between restraint sessions, and footshock on PND 27 (6×0.5 mA, 0.5 s footshocks over 3 min in a 30 cm ×25 cm ×32 cm shock chamber (Coulbourn Instruments, PA)). This protocol has previously been shown to produce enhanced anxiety responses and depressive-like behaviours in rodent models [12], [13]. The rats were then left undisturbed in their home cages in groups of 4–5 until testing. As adults, the ELS rats (n = 8) were exposed to fear conditioning (as PG) and fMRI as outlined above and their activation patterns were compared to control (CON) rats that had not received ELS.

## Results

### fMRI activation maps in response to Conditioned Stimulus

**Figure 3:**
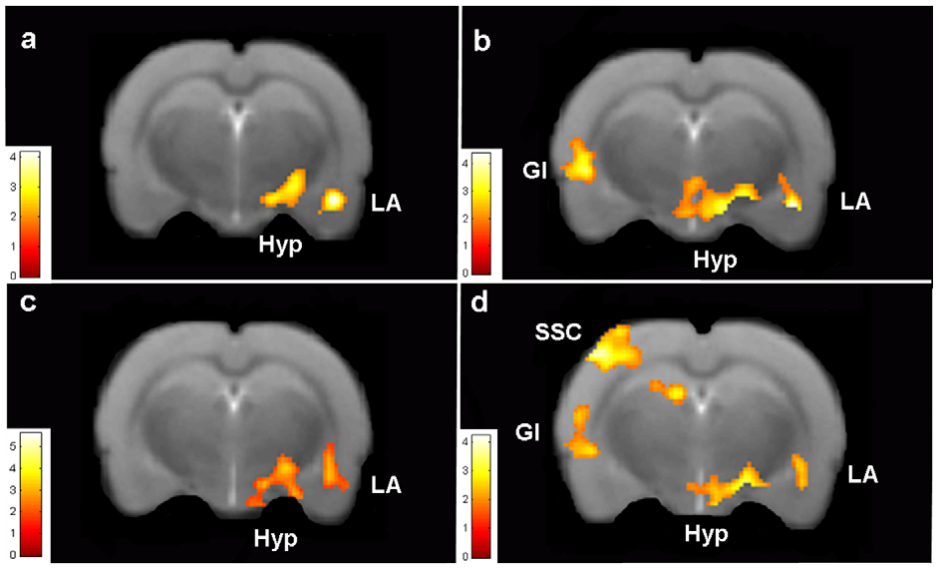
Brain activation maps in response to a fear conditioned stimulus. (a) Activation in PG in response to CS presentation is observed in right lateral amygdala (LA) and hypothalamus (Hyp). (b) Comparison of response between groups (PG:UG) confirms LA, Hyp and granular insular cortex (GI) activation in PG in response to the CS presentation. Extinction modeling of (c) the response across CS presentations within the PG or (d) between PG and UG groups reveals right LA, Hyp, GI and somatosensory cortex activation (SSC). PG, n = 14; UG, n = 10.

Analysis of neural responses (BOLD activation) in animals from the PG during the first CS presentation in the scanner compared to the rest periods revealed activation of a neural system implicated in fear processing, comprising the right lateral amygdala (LA), hypothalamus (Hyp), somatosensory cortex (SSC) and granular insular cortex (GI) (Figure 3a; Table 2). In contrast the UG did not show activation of any of these fear-associated brain regions during the CS presentation (Table 2). Direct comparison of activation to the first CS in the PG compared to the UG confirmed greater activation in the PG of the right LA, Hyp, GI, left SSC and ventral pallidum (Figure 3b, Table 2). Analysis of brain responses across the three CS presentations revealed evidence of amygdala habituation over time, consistent with previous human MRI studies [7], [8] (Figure 3c–d; Table 3).

**Table 2:** Brain activation during first CS presentation versus baseline.

	KE	T	Region
**PG within group (First CS> Baseline)**			
Whole brain analysis	495	3.18	R lateral hypothalamus R lateral amygdala L somatosensory cortex R + L granular insular cortex
Lateral amygdala mask	117	4.17	R lateral amygdala*
**UG within group (First CS> Baseline)**			
Whole brain analysis	12020	15.43	Peak R subiculum extends to adjacent cortex

	KE	T	Region
**PG>UG (First CS> Baseline)**			
Whole brain analysis	1781	4.37	R lateral amygdala Hypothalamus (PVN)
	1609	3.40	L Somatosensory cortex
	676	3.15	Ventral pallidum
Lateral amygdala mask	70	4.37	R lateral amygdala*
**UG>PG (First CS> Baseline)**			
Whole brain analysis	5611	4.38	Medial geniculate nucleus
	593	3.62	Sensory cortex

Significant regional brain activation (BOLD responses) to the first CS presentation compared to baseline. PG  =  paired group, UG  =  unpaired group. KE  =  cluster extent in voxels. T  =  peak voxel t-statistic. Clusters reaching *P*_corrected_ <0.05 are shown.*  =  significant (*P*_corrected_ <0.05) within lateral amygdala ROI. R =  right, L =  left, PVN =  paraventricular nucleus. PG, n =  14; UG, n = 10.

**Table 3:** Brain activation across extinction.

	KE	T	Region
**PG within group analysis (First CS >Last CS)**			
Whole brain analysis	826	5.61	R lateral amygdala
	582	3.48	Hypothalamus
Lateral amygdala mask	147	3.35	R lateral amygdala*
**PG within group analysis (Last CS> First CS)**			
Whole brain analysis	661	5	Thalamus (extending to central amygdala)
	1589	4.19	Sensory cortex
	1242	3.7	Ventral subiculum, midbrain
	444	3.09	Ventral pallidum
**Between Group Analysis**			
**PG>UG (First CS>Last CS)**			
Whole brain analysis	1211	4.21	Sensory cortex
	485	4.11	R lateral amygdala
	1295	3.99	Granular insular cortex
	825	3.26	Hypothalamus
Lateral amygdala mask	91	3.35	R lateral amygdala*
**PG>UG (Last CS> First CS)**			
	1542	3.97	RSG
	1221	3.9	Ventral subiculum includes medial geniculate nucleus
	457	3.89	Thalamus

Significant regional brain activation (BOLD responses) to the first CS presentation compared to last CS presentation and the reverse. PG  =  paired group, UG  =  unpaired group. KE  =  cluster extent in voxels. T  =  peak voxel t-statistic. Clusters reaching *P*_corrected_ <0.05 are shown.*  =  significant (*P*_corrected_ <0.05) within lateral amygdala ROI. R =  right, L =  left, RSG =  retrospenial granular cortex. PG, n =  14; UG, n = 10.

### Early Life Stress causes increased activation of fear networks

**Figure 4:**
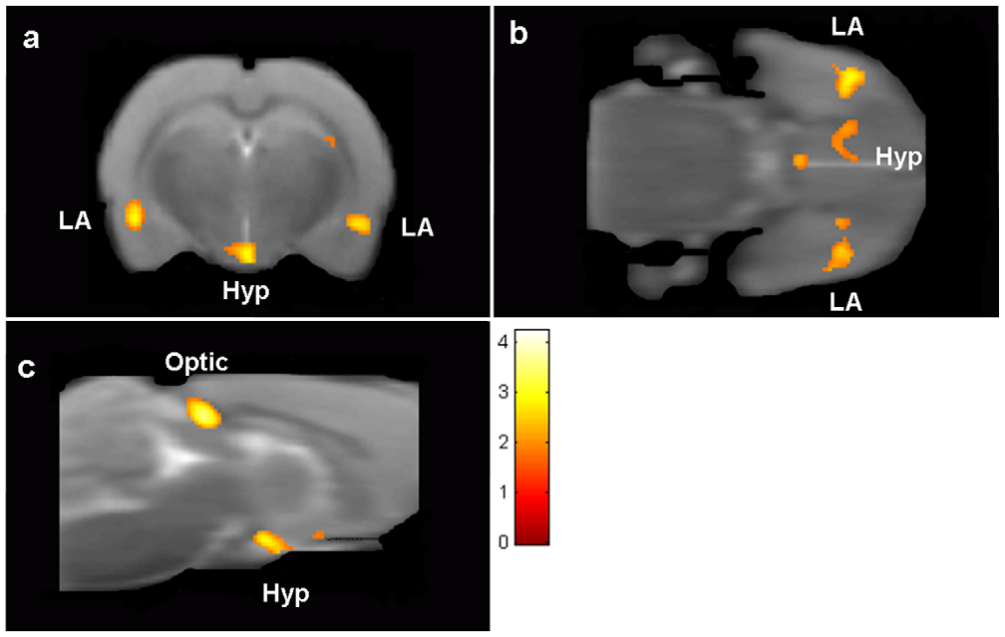
Consequence of early life stress on fear associated brain activation. Greater activation in response to CS presentation is observed in right and left lateral amygdalae (LA), hypothalamus (Hyp) and optic grey (optic) when ELS is compared to CON. (a) Coronal, (b) axial and (c) sagittal sections of rat brain. Control, n = 14; ELS, n = 8.

We next investigated fear circuitry activation following early life stress (ELS). Analysis of neural responses during the presentation of the fear-associated CS in the ELS group revealed activation of a neural system comprising the left LA, SSC, retrosplenial granular cortex, ventral pallidum and caudate putamen during the first CS presentation compared to the rest periods (Table 4). Comparison of the ELS group to control animals showed greater activation of fear circuitry including both left and right amygdalae and hypothalamus in the ELS group compared to the CON group during the first CS presentation (Figure 4 and Table 4). These results demonstrate that ELS increases amygdala activation during adulthood, consistent with human studies [14]–[17].

**Table 4:** Effects of early life stress on brain activation.

	KE	T	Region
**ELS Within Group Analysis (First CS>Baseline)**			
Whole brain analysis	684	8.62	L ventral pallidum/cortex
	1097	6.36	RSG/dorsal subiculum
	725	5.66	R somatosensory cortex
	793	5.42	Ventral pallidum extending to L lateral amygdala
	662	4.98	R caudate putamen
**Between Group Analysis**			
**ELS>CON (First CS>Baseline)**			
Whole brain analysis	3808	4.23	Somatosensory cortex extending to thalamus and R lateral amygdale later latere lateral amygdala
	1107	4.04	RSG/Dorsal subiculum
	919	3.63	L ventral pallidum
	2447	3.08	L central and lateral amygdala, lateral hypothalamus
**CON>ELS (First CS>Baseline)**			
Whole brain analysis	541	3.15	R subiculum extending to brainstem

Within and between group analysis comparing BOLD response to first CS presentation over baseline in rats previously exposed to early life stress (ELS) compared to unstressed controls (CON). KE  =  cluster extent in voxels. T  =  peak voxel t-statistic. Clusters reaching *P*_corrected_ <0.05 are shown. R =  right, L =  left, RSG =  retrospenial granular cortex. CON, n =  14; ELS, n = 8.

## Discussion

Here we have developed a novel fMRI paradigm for examining neural activation in awake rodents processing a learned emotional stimulus. Using this method we demonstrate activation of fear-related circuitry, including the amygdala and hypothalamus, to an aversively conditioned CS. We also show that this fear response is exacerbated in an animal model of human affective disorders based on prior exposure to ELS. This represents the first demonstration of the delineation of regional brain function in response to a learnt association in awake rodents using fMRI, opening the door to the use of fMRI for translational research into human affective and neuropsychiatric disorders.

In order to demonstrate specific changes in neural activation related to fear learning, we used a tightly controlled fear conditioning paradigm. We were able to show that only rats in which the CS was specifically paired with the US, leading to a conditioned association, demonstrated increased activation of the right LA, Hyp, SSC and GI in response to re-exposure to the CS. In contrast no such responses were seen in animals exposed to the same stimuli in an unpaired (non-contingent) manner. The LA is known to be a key brain region involved in the formation of fear associations and emotional learning, whilst the Hyp is central to the mediation of behavioural responses to fear-associated stimuli [22], [23]. The GI and SSC are constituents of pain pathways, which are recruited into the conditioned network in response to fear-associated CS [24], [25]. These results closely parallel previous human fear conditioning fMRI studies [8] and also indicate an unexpected, but not unrecognised, lateralization of amygdala activation during the expression of conditioned fear [14], [26].

We additionally investigated whether our fMRI paradigm was sensitive to the effects of early life stress. ELS has previously been shown to result in increased anxiety and depression-like behaviour in rodent models [11]–[13]. In our current study we show that pre-pubertal ELS increases bilateral LA and Hyp activation to fear-associated stimuli in adulthood. This demonstrates the utility of rodent fMRI for investigating the aetiology and treatment of affective disorders using methods that can be directly translated into human imaging studies.

Although previous studies have used fMRI in animals, and the behavioural and neural circuitry of fear conditioning are well validated, the novel combination of these techniques in the present study allows a system wide analysis of brain activation during such cognitive and emotional responses. These findings demonstrate that it is possible to apply fMRI to the analysis of regional brain activation during learnt tasks in awake rodents, paving the way to translational investigations of the effects of experimental manipulations and of the neural basis of fMRI signal changes that would be difficult, if not impossible, to achieve using human studies alone.
